# Modeling the biomechanics of fetal movements

**DOI:** 10.1007/s10237-015-0738-1

**Published:** 2015-11-03

**Authors:** Stefaan W. Verbruggen, Jessica H. W. Loo, Tayyib T. A. Hayat, Joseph V. Hajnal, Mary A. Rutherford, Andrew T. M. Phillips, Niamh C. Nowlan

**Affiliations:** Department of Bioengineering, Imperial College London, London, SW7 2AZ UK; Division of Imaging Sciences, Department of Biomedical Engineering, Kings College London, London, UK; Division of Imaging Sciences, Department of Biomedical Engineering & Centre for the Developing Brain, Kings College London, London, UK; Division of Imaging Sciences, Department of Perinatal Imaging and Health & Centre for the Developing Brain, Kings College London, London, UK; Structural Biomechanics, Department of Civil and Environmental Engineering, Imperial College London, London, UK

**Keywords:** Musculoskeletal development, Joint biomechanics, Cine MRI, Developmental dysplasia of the hip, Computational model

## Abstract

Fetal movements in the uterus are a natural part of development and are known to play an important role in normal musculoskeletal development. However, very little is known about the biomechanical stimuli that arise during movements in utero, despite these stimuli being crucial to normal bone and joint formation. Therefore, the objective of this study was to create a series of computational steps by which the forces generated during a kick in utero could be predicted from clinically observed fetal movements using novel cine-MRI data of three fetuses, aged 20–22 weeks. A custom tracking software was designed to characterize the movements of joints in utero, and average uterus deflection of $6.95 \pm 0.41$ mm due to kicking was calculated. These observed displacements provided boundary conditions for a finite element model of the uterine environment, predicting an average reaction force of $0.52 \pm 0.15$ N generated by a kick against the uterine wall. Finally, these data were applied as inputs for a musculoskeletal model of a fetal kick, resulting in predicted maximum forces in the muscles surrounding the hip joint of approximately 8 N, while higher maximum forces of approximately 21 N were predicted for the muscles surrounding the knee joint. This study provides a novel insight into the closed mechanical environment of the uterus, with an innovative method allowing elucidation of the biomechanical interaction of the developing fetus with its surroundings.

## Introduction

Physical movements in the uterus are a normal part of fetal development, with most movements observable by ten gestational weeks using ultrasound (Vries and Fong 2006). These movement patterns can comprise whole-body movements, limb movements, breathing movements and stretching (Vries et al. 1982), with maternal sensation of these movements usually beginning at 16–18 weeks (Vries et al. 1982). It has been found that fetal movement can be a significant indicator of fetal health, with studies showing that decreased fetal movement may precede fetal demise/stillbirths (Efkarpidis et al. 2004; Whitworth et al. 2011). Similarly, maternal perception of decreased fetal movements has been linked to poor outcomes at birth, such as preterm or low-birth-weight babies, in 22–25 % of cases (Dutton 2012; O’Sullivan et al. 2009). In addition to being a guide to general fetal health, fetal movements are particularly important for musculoskeletal development (reviewed in Nowlan 2015), as indicated in cases of decreased fetal movement due to neuromuscular disorders presenting various skeletal abnormalities such as multiple joint fusions, craniofacial malformations and thin hypo-mineralized bones (Aronsson et al. 1994; Rodríguez et al. 1988a, b).

Indeed, direct evidence of the role of mechanical stimulation has been observed in animal models, with similar joint and bone tissue abnormalities resulting from muscle immobilisation in chick embryos, and in mouse embryos with reduced or immobile muscles (Kahn 2009; Nowlan et al. 2010a, b, 2014; Roddy et al. 2011). A further study of muscle-less mouse embryos has identified key developmental regulatory genes which are down-regulated in the absence of mechanical stimuli (Rolfe 2014). Therefore, mechanical forces generated by fetal movement are important for prenatal musculoskeletal development, and this is particularly true for joint shape (Kahn 2009; Nowlan et al. 2014). A relatively common example of abnormal joint shape in human babies is developmental dysplasia of the hip (DDH) (Leck 2000), which occurs when the joint formed by the femoral head and the acetabulum is unstable, malformed or dislocated (Weinstein 1987). Significantly, joint shape abnormalities such as DDH lead to increased risk of osteoarthritis in later life (Muller and Seddon 1953). While genetic influences exist, such as female gender and positive family history, major environmental risk factors for DDH include fetal breech position (Muller and Seddon 1953), low amniotic fluid volume (oligohydramnios) (Hinderaker et al. 1994) and neuromuscular disorders (Homer and Hickson 2000). The common element in each of these cases is that the movement of the fetus in the uterus is restricted, indicating that a link may exist between fetal movement and abnormal joint development (Nowlan 2015). However, as the uterus is a closed system that is difficult to directly observe without interfering with its mechanical environment, the biomechanics of fetal movements remain poorly understood.

Recently developed cine-MRI techniques provide a novel ability to simultaneously view movements of the fetal limbs, head and trunk, allowing direct observation of whole-body fetal movements (Guo et al. 2006; Hayat et al. 2011). Separately, computational finite element analysis is often used to characterize complex biomechanical environments, such as the hip joint (Phillips et al. 2007). However, to date, application of finite element analysis to pregnancy has focussed on either the effects of the pregnancy on the surrounding tissues, such as those of the cervix (House et al. 2012, 2013), or the effects of the external mechanical environment on the fetus, such as during labor or vehicle collisions (Lapeer and Prager 2001; Serpil Acar and Lopik 2009). Indeed, MRI techniques have recently been employed to generate accurate three-dimensional finite element models of the uterine environment during pregnancy (Fernandez et al. 2015). Musculoskeletal modeling techniques are used to estimate joint forces during dynamic activities in adult humans (Modenese et al. 2011, 2013; Modenese and Phillips 2012), but these methods have never before been applied to the fetal skeleton.

Therefore, the objective of this research is to employ computational techniques to predict the mechanical forces that occur due to clinically observed fetal movements, with particular emphasis on the hip joint. This will enable a better understanding of the biomechanical importance of fetal kicks, and provide a novel method to investigate skeletal abnormalities such as DDH.

## Materials and methods

**Fig. 1:**
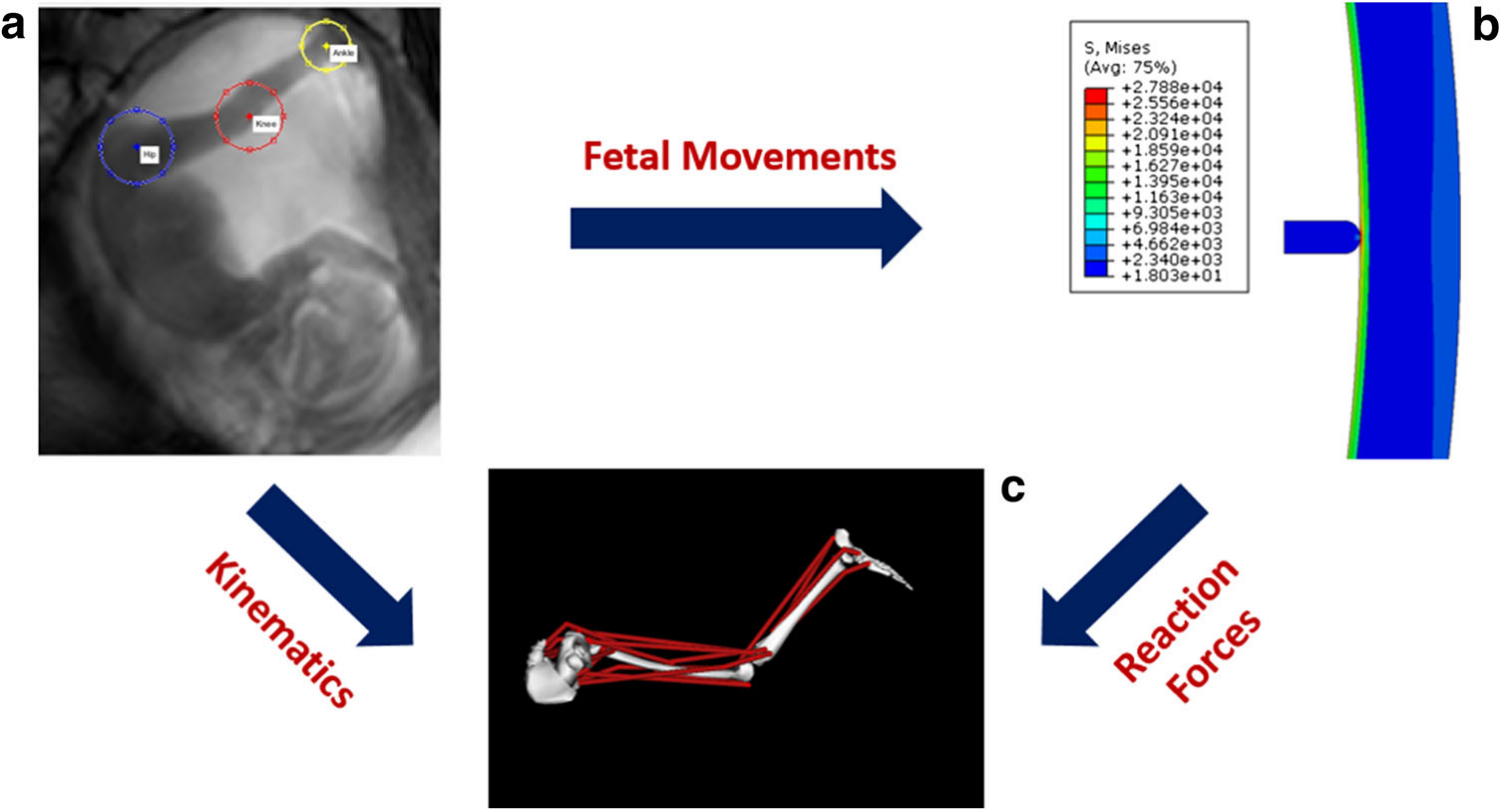
Schematic of the relationship between the three methods employed to investigate fetal biomechanics: **a** tracking of fetal joint movements, **b** FE model of effect of displacement on the uterus (stress shown) and **c** musculoskeletal model to predict intramuscular force

The development of models to investigate fetal movements required three separate steps: (1) tracking of joint displacements within the uterus during kicking, (2) calculation of the reaction forces resulting from these displacements and (3) prediction of the intramuscular forces required to generate the observed displacements and forces. The relationship between these three steps is illustrated in Fig. 1, and the methods are described in detail in this section.

### Tracking software

In order to elucidate the displacement of individual joints, as well as the deflection of the uterine wall caused by fetal kicking, a custom-designed script was developed using MATLAB R2014b (Mathworks, UK). This software allowed automatic tracking of joint displacements during fetal kicking, measured from novel cine-MRI data capturing fetal movements in utero (Hayat et al. 2011).

Images were obtained from archived data at the Imperial College School of Medicine (Hammersmith Hospital, London, UK). Women were either referred for clinical reasons or volunteered for a research scan, with all images being acquired after 20-week gestation. All women gave written consent to research (Hammersmith Hospital Research Ethics Committee) and were scanned in the left lateral tilt position on a 1.5-Tesla Philips Achieva scanner (Phillips Healthcare, Best, Netherlands). Cine images were acquired using an optimized balanced steady-state free precession (bSSFP) sequence with the following parameters: flip angle, $60^{\circ }$; FOV, $50\;\mathrm{cm}^{2}$; TR/TE, 3.2/1.59 ms; voxel size, 2.2 $\times$ 2.2 mm; partial Fourier, 62.5 %; SENSE factor, 2; SAR, 2 W/kg; section acquisition time, 0.303 s (Hayat et al. 2011). Scans of three different fetuses were examined, at gestational ages of 20, 21 and 22 weeks. The fetuses had normal brain MRI scans and were normal at subsequent neurodevelopmental follow-up. Scans were taken with a section thickness of 30–40 mm preventing loss of data in the event of slight out-of-plane movements (Hayat et al. 2011). Kicking sequences were selected from longer scans during which frequent spontaneous fetal movements occurred. The kicks were chosen based on simple in-plane extension of both the hip and knee joints, such that the foot is brought into sustained contact with the uterine wall. Movements selected were consistent and comparable between different scans. ImageJ analysis software (Schneider et al. 2012) was used to measure the distance between the hip and knee joints (referred to here as femur length), and the knee and ankle joints (referred to here as tibia length), providing data for scaling the musculoskeletal models. Additionally, the uterine dimensions were measured, assuming an elliptical shape with a major and a minor axis. A series of images was analyzed for each fetus, capturing the kick and contact with the uterine wall, up to the point of greatest deflection of the wall. These kicks lasted 3.0, 2.0 and 3.3 s for Fetus A, Fetus B and Fetus C, respectively.

**Fig. 2:**
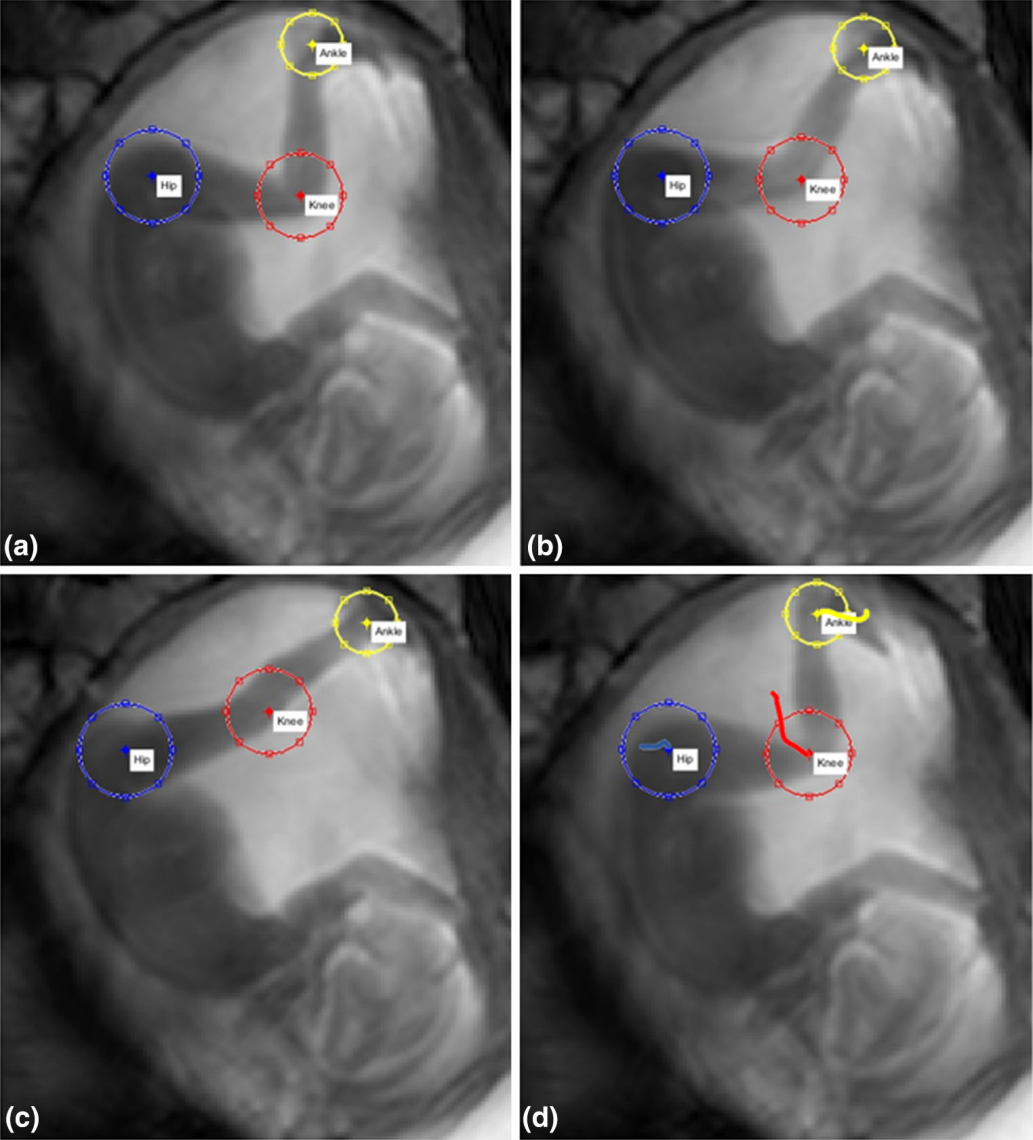
Successive frames (**a**-**c**) of cine-MRI scans, with (**d**) showing paths of displacement from automatic tracking of hip, knee and ankle joints using custom software

To track the joint displacements, the hip, knee and ankle were manually selected, with these regions serving as initial templates for the scan. Independently of the ImageJ measurements, the femur and tibia lengths calculated by the tracking software were maintained throughout the sequence, with a change in length of $\pm$10 % allowed to account for slight out-of-plane movement. In each successive scan in the cine-MRI series, the hip was identified using template matching (see Fig. 2). Possible locations of the knee were then identified using the femur length and the maximum likely movement of the knee compared to the previous frame. Within the possible location space of the knee, template matching was used to determine its position. Once the knee joint location had been identified, the process was repeated to locate the ankle joint.

This entire process was then repeated for each successive frame, with the templates accumulated and updated as the tracking progressed. Thus, the templates from all previous frames were used, with weighting applied to give recent frames more importance as the representation of the joint is more similar. The automatic tracking software is accurate in approximately 95 % of cases compared to manual selection by an experienced human operator, and as the template matching is based on templates accumulated from previous frames, the process is fully repeatable. The uterus deflection was recorded as the translational displacement of the ankle joint while in contact with the uterine wall.

### Finite element modeling

**Fig. 3:**
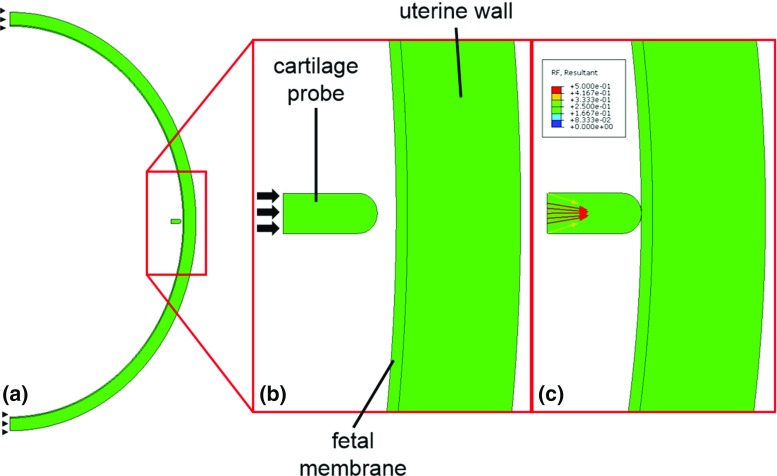
**a** Diagram of symmetry boundary conditions in FE model of uterus, **b** diagram showing application of displacement boundary condition to the fetal cartilage probe and **c** reaction force magnitudes (in newtons) and vectors resulting from uterus displacement

Finite element (FE) simulations were conducted to investigate the reaction force resulting from the displacement of the uterus wall observed using the tracking software. Three computational FE models of the uterine environment were generated, with the uterus modeled as an ellipse using dimensions taken from each scan. The uterine wall comprised a 0.6-mm-thick fetal membrane (Buerzle 2013) and a 6-mm-thick layer of uterine muscle (Sokolowski et al. 2010). The fetal membrane was assumed to have an elastic modulus of 7.53 MPa, a stiffness that was extrapolated to 20 weeks based on previous testing of preterm and term membranes (Benson-Martin 2006). An elastic modulus of 586 kPa was assumed for the uterus muscular tissue, converted from 85 psi reported in the available literature on pregnant uterine material properties (Pearsall and Roberts 1978). Half of the uterus environment was modeled, with symmetry boundary conditions applied at the boundaries (see Fig. 3a). In order to simulate a fetal kick, a probe was generated of the same diameter as the fetal foot, to which the observed displacement from the tracking step was applied as ramped, static loading. Initially, the geometries of all components were as described above, with deformation occurring once the fetal foot was brought into contact with the fetal membrane. While the full motion sequence of each kick was tracked using the tracking software, the FE modeling was confined to the time during which the foot was in contact with the uterus wall. The probe was assumed to have mechanical properties similar to fetal cartilage and was assigned an elastic modulus of 1.1 MPa (Tanck et al. 2004), while contact between the probe and the fetal membrane was assumed to be frictionless due to their smooth surfaces and amniotic fluid acting to prevent friction between the surfaces. Furthermore, a sensitivity analysis was performed to determine the effect of the cartilage material properties on reaction forces, which found negligible changes of approximately $\pm \; 0.8\,\%$ in the reaction force resulting from a doubling or halving of the elastic modulus. All components were meshed using four-noded quadrilateral plane stress shell elements (CPS4). Contact was made at the midpoint of the elliptical geometry, both because this was analogous to the region kicked by the fetuses in the scans and in order to avoid edge effects from the boundary conditions. All materials were assumed to be linear elastic and isotropic in nature, with a Poisson’s ratio of 0.49 for the fetal cartilage probe (Armstrong et al. 1984; Carter and Beaupré 1999; Wong et al. 2000), and 0.4 for the fetal membrane and uterine muscle (Serpil Acar and Lopik 2009). Finally, it was assumed that there were no external forces acting on the system and that the primary resistance came from the uterine wall and fetal membrane.

### Model validation

**Fig. 4:**
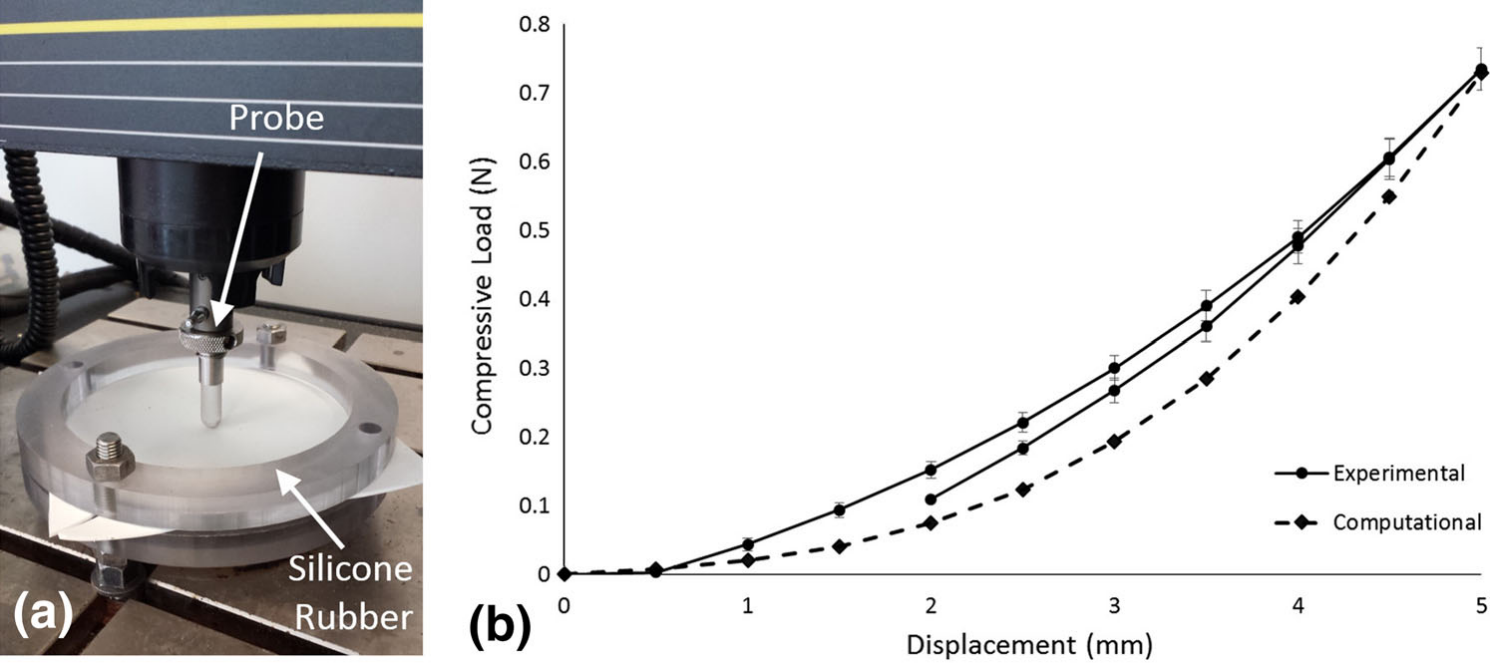
**a** Image of experimental setup showing Instron machine, probe and silicone rubber sheet, and **b** graph comparing average of experimental forces with forces predicted computationally (*error bars* show standard deviation, *arrows* indicate loading and unloading curves)

In order to determine whether a 2D FE model could accurately predict the reaction forces resulting from a fetal kick, an experimental setup was designed to compare with our computational models. This setup is shown in Fig. 4a and comprised a 16 $\times$ 16 cm silicone rubber sheet (RS Components, Northants, UK) constrained concentrically by two 1.5-cm-thick 3D-printed ABS (Objet Ltd., Stratasys, MN, USA) circular clamps. An Instron 5866 (Instron, MA, USA) mechanical testing machine was fitted with a round-ended, 10- mm-diameter 3D-printed ABS (Objet Ltd., Stratasys, MN, USA) cylindrical probe, and was used to apply a displacement of 5 mm to the surface of the silicone rubber sheet at a rate of 5 mm/s under displacement control, before then removing the displacement. This test was repeated three times each for three samples, with the average maximum force found to be 0.735 N.

These results were compared to a 2D FE model of a probe being pressed into a sheet, using the same dimensions as those of the 3D-printed experimental components. The ABS parts were assumed to have an elastic modulus of 2.6 GPa with a Poisson’s ratio of 0.3, while the silicone rubber was assigned an elastic modulus of 10.3 MPa and a Poisson’s ratio of 0.49, with these material properties provided by the respective manufacturers. The silicone rubber sheet was fully constrained at each end, while a displacement boundary condition of 5 mm was applied to the probe. Contact between the probe and the sheet was assumed to be frictionless. The maximum total reaction force predicted was 0.729 N, and these results are shown in Fig. 4b alongside the average experimental results. It can be seen that a close correlation exists between the experimentally observed forces and those predicted computationally, over multiple time points.

**Table 1:** Table of the different gestational ages, femur and tibia lengths, uterine major and minor axes, maximum kick-induced uterus deflection and maximum kick-induced nodal reaction forces for each fetus investigated, expressed individually and as an average

	Gestational age (weeks)	Femur length (mm)	Tibia length (mm)	Uterine major axis (mm)	Uterine minor axis (mm)	Maximum displacement (mm)	Maximum nodal reaction force (N)
Fetus A	20	51.02	54.58	160.12	155.69	6.40	0.72
Fetus B	21	58.34	60.86	223.34	156.88	7.37	0.33
Fetus C	22	52.41	59.81	185.09	175.43	7.07	0.51
Average	21 $\pm$ 0.82	53.92 $\pm$ 3.18	58.41 $\pm$ 2.75	189.51 $\pm$ 26.00	162.67 $\pm$ 9.03	6.95 $\pm$ 0.41	0.52 $\pm$ 0. 16

### Musculoskeletal modeling

In order to determine the muscle forces required to generate the observed movement and reaction forces for each fetus, musculoskeletal models of the fetal leg were generated in OpenSim (Delp 2007). The model was based on the 3DGaitModel2354 model, with all bodies removed except the right pelvis, femur, tibia, talus, calcaneus and toes, scaled to the dimensions of each fetus using the lengths calculated in ImageJ. A total of 18 muscles were included in the model, with the muscle paths enhanced via points and wrapping surfaces where the muscles were allowed to slide without friction. The maximum isometric force, force-velocity and length-force restrictions were unchanged from the original model. The model included five joints, where the hip was modeled as a ball and socket joint, the tibio-femoral joint was represented as a hinge and the ankle joint comprised the talocrural and the subtalar joints (with these ankle joints locked). Movement was confined to a plane as the data from the scans were two-dimensional, with movement constrained in the z-direction.

The displacement data from the tracking software were then applied to the joint markers, and the reaction forces from the FE models were applied at the calcaneus (heel bone) of the fetal foot, with these two datasets acting as boundary conditions for the models (see schematic in Fig. 1). An inverse kinematics step was performed to characterize the fetal movement using the tracking data, followed by an inverse dynamics step to determine the intramuscular forces required to generate the movement. The effect of gravity was neglected as the fetus and amniotic fluid have similar specific gravities (1.055 and 1.009, respectively) (Wood 1970). Furthermore, as all skeletal muscles have developed by approximately 8 weeks (Bardeen and Lewis 1901), it was assumed that each muscle was present and active as it would be postnatally. Finally, a quadratic static optimization calculation was performed, whereby OpenSim predicted the most likely muscle activation patterns and forces that would result in the observed movement and reaction forces. Reserve actuators acting on the six degrees of freedom of the pelvis with respect to the ground reference system were defined in order to compensate for the dynamic contributions of the missing torso and contralateral leg during the static optimization process. The muscles were segregated into two groups, by proximity of muscular origin to the hip or knee joint.

## Results

The average lengths of the femur and tibia were 53.92 $\pm$ 3.18 and 58.413 $\pm$ 2.75 mm, respectively, with individual measurements shown in Table 1. Similarly, the major and minor axes of the uterus are shown, with average values of 189.51 $\pm$ 26.00 and 162.67 $\pm$ 9.03 mm, respectively. The average maximum displacement of the uterine wall was found to be 6.95 $\pm$ 0.41 mm, with the individual results for each of the three fetuses shown in Table 1.

This displacement, when applied to the uterine wall using the fetal cartilage probe in the FE model, resulted in an average maximum nodal reaction force of 0.52 $\pm$ 0.15 N. The maximum nodal reaction force was recorded at the location of the applied boundary condition due to equal and opposite reactions, as shown in Fig. 3c. The individual reaction forces for each fetus are shown in Table 1.

**Fig. 5:**
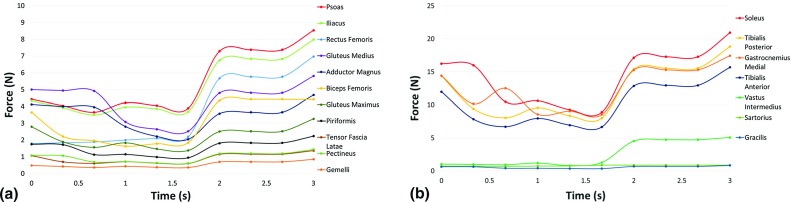
Graph showing intramuscular forces for the major muscles surrounding **a** the hip joint and **b** the knee joint during a fetal kick from Fetus A

**Fig. 6:**
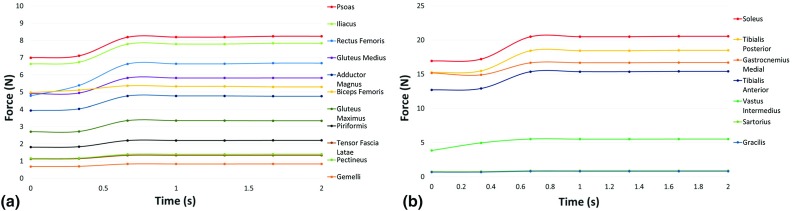
Graph showing intramuscular forces for the major muscles surrounding **a** the hip joint and **b** the knee joint during a fetal kick from Fetus B

**Fig. 7:**
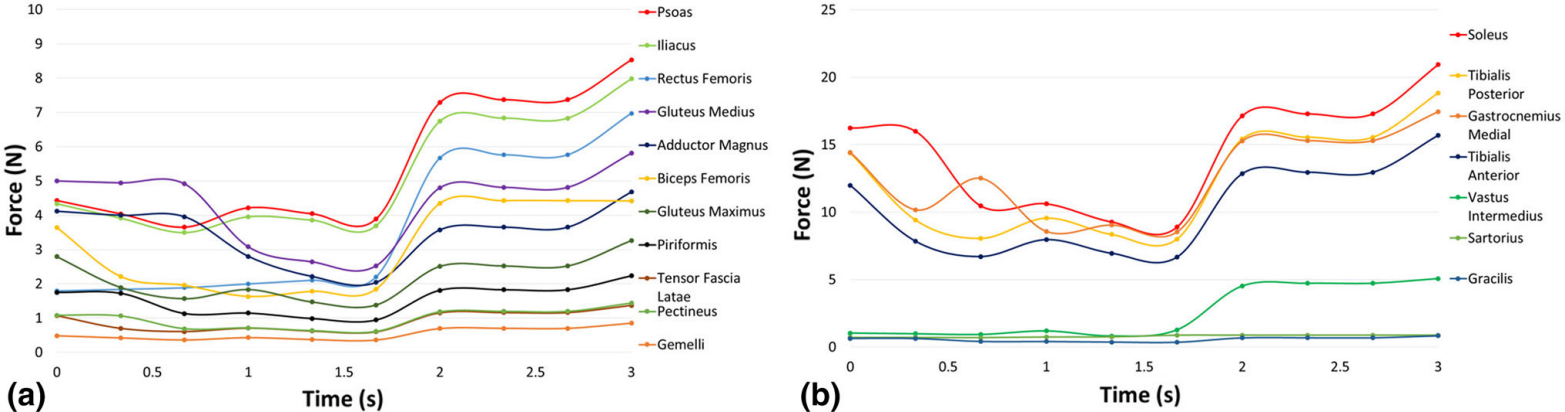
Graph showing intramuscular forces for the major muscles surrounding **a** the hip joint and **b** the knee joint during a fetal kick from Fetus C

The joint displacements and total reaction force on the fetal foot derived from the tracking and FE steps, when applied as boundary conditions in the OpenSim musculoskeletal model, resulted in predicted intramuscular forces for muscles surrounding the hip joint and the knee joint. These are shown for Fetus A, Fetus B and Fetus C in Figs. 5, 6 and 7, respectively. The maximum intramuscular force generated by each muscle at the hip joint is listed for each fetus investigated in Table 2. Regarding the hip joint, it can be seen that the greatest maximum forces were produced by the iliacus and psoas muscles (8.17 $\pm$ 0.38 and 8.64 $\pm$ 0.37 N, respectively). On average, similar maximum forces were produced by the rectus femoris, gluteus medius, adductor magnus and biceps femoris muscles (6.81 $\pm$ 0.12, 5.77 $\pm$ 0.08, 5.41 $\pm$ 0.98 and 4.52 $\pm$ 0.60 N, respectively). The lowest maximum forces were predicted for the gemelli muscles (0.086 $\pm$ 0.03 N).

Similarly, the maximum intramuscular forces generated by each muscle at the knee joint are listed for each fetus investigated in Table 3. The forces generated by the muscles surrounding the knee joint were much greater, with the greatest intramuscular forces produced by the soleus muscle (21.18 $\pm$ 0.64 N). The average maximum forces were similarly high for the tibialis posterior, tibialis anterior and gastrocnemius medial muscles (19.06 $\pm$ 0.58, 15.88 $\pm$ 0.48 and 17.69 $\pm$ 0.92 N, respectively). Finally, the lowest maximum forces were generated by the gracilis muscle (0.85 $\pm$ 0.05 N).

**Table 2:** The maximum intramuscular forces, in newtons, generated by each major muscle surrounding the hip joint shown for each fetus, expressed individually and as an average

	Psoas	Iliacus	Rectus femoris	Gluteus medius	Adductor magnus	Biceps femoris	Gluteus maximus	Piriformis
Fetus A	8.54	7.97	6.97	5.81	4.68	4.42	3.26	2.23
Fetus B	8.24	7.83	6.68	5.82	4.76	5.29	3.34	2.20
Fetus C	9.13	8.69	6.77	5.64	6.80	3.83	3.17	2.35
Average	8.64 $\pm$ 0.37	8.17 $\pm$ 0.38	6.81 $\pm$ 0.12	5.77 $\pm$ 0.08	5.41 $\pm$ 0.98	4.52 $\pm$ 0.60	3.26 $\pm$ 0.71	2.26 $\pm$ 0.06

**Table 3:** The maximum intramuscular forces, in newtons, generated by each major muscle surrounding the knee joint shown for each fetus, expressed individually and as an average

	Soleus	Tibialis posterior	Gastrocnemius medial	Tibialis anterior	Vastus intermedius
Fetus A	20.93	18.84	17.44	15.69	5.08
Fetus B	20.55	18.49	16.70	15.41	5.53
Fetus C	22.06	19.86	18.91	16.54	4.64
Average	21.18 $\pm$ 0.64	19.06 $\pm$ 0.58	17.69 $\pm$ 0.92	15.88 $\pm$ 0.48	5.09 $\pm$ 0.36

## Discussion

This study provides a novel insight into the biomechanical environment of the uterus, through the use of cine-MRI data of fetal movements and computational modeling techniques. While tracking joint movements during fetal kicks, we observed an average displacement of the uterus wall of 6.95 $\pm$ 0.41 mm, with these kicks generating an average reaction force of 0.52 $\pm$ 0.15 N. Thus, we predict for the first time the force generated by individual muscles during kicking movements in the uterus, ranging from 0.85 $\pm$ 0.04 N in the gracilis to 21.18 $\pm$ 0.64 N in the soleus. These models shed light on the biomechanical stimuli experienced in the uterus, indicating the muscles that play a prominent role in both hip and knee joint movements during fetal kicking.

Poor existing knowledge of the mechanical environment of the uterus necessitated a number of assumptions in the development of these models. Firstly, while the cine-MRI technique provides novel data of movements in utero, scans are captured as a thick 2D slice through the uterus. Therefore, while both the tracking software and FE models captured 2D planar movements, these inputs predicted muscle forces in 3D in OpenSim. However, this is an inherent property of OpenSim musculoskeletal modeling and these MRI scans represent the best available method for observing fetal movements. Also, as it is not possible to validate the musculoskeletal model using EMG in utero and there is no available data in the literature for fetal muscles, the maximum isometric force, force-velocity and length force restrictions were set to the same as those of an adult human model, which have been developed by collecting datasets from anatomical studies (Arnold et al. 2010). Additionally, while nonlinear material properties are available for fetal membrane tissue (Buerzle 2013), these data are for late gestational ages ($>$37 weeks) and, therefore, are likely different to that experienced throughout pregnancy. Furthermore, an elastic modulus of 586 kPa was assumed for the uterus based on studies of tissue excised during hysterectomy, which could have different mechanical properties from in vivo tissue during pregnancy (Pearsall and Roberts 1978). Similarly, previous studies to characterize the mechanical properties of the fetal membrane were tensile tests performed in controlled laboratory conditions, which differ greatly from in vivo conditions (Benson-Martin 2006). External forces from outside the uterine wall are assumed to be balanced by the intrauterine pressure, and so both are excluded from these analyses. Additionally, drag forces due to movement through amniotic fluid are neglected, as both ends of the limb are in contact with the uterus during the analysis. It can be seen that, although the time histories differ, in each fetus the intramuscular forces ramp up to similar maximum forces on complete extension of the leg. The similarity of this behavior between different fetuses is an indication of the robustness of the modeling process, and while the absolute values of the forces predicted here may not be precise, this methodology will enable us to compare between different environmental factors.

It is interesting to note that the muscles surrounding the tibia and affecting the knee joint generate significantly greater forces than at the hip joint ($\sim$16–21 N vs. $\sim$5–8 N). Four muscles in particular appear to play an important role in fetal extension kick movements in utero, with the soleus, tibialis posterior, tibialis anterior and gastrocnemius medial each generating relatively large amounts of force at the knee joint. In contrast, many of the muscles of the upper leg appear to contribute much less to the kicking movement. Interestingly, a study of spontaneous free leg movements in new-born infants has shown little posterior muscle activation during extension, in contrast to our observations (Thelen and Fisher 1983). This may be due to the lack of resistance provided by a surface, such as the uterine wall or the ground. Indeed, a study of one individual found greater use of posterior muscles (gastrocnemius and biceps femoris) compared to anterior muscles (tibialis anterior and rectus femoris) when learning to walk, with this predominance reducing over time (between the ages of three weeks and seven years) (Okamoto et al. 2003). Additionally, the high forces in the iliacus and psoas muscles may arise due to the fact that the fetus must counterintuitively reduce the angle between the torso and the hip during kicking, due to the restricted space in the uterus. As all of these muscles act in three dimensions and in multiple different directions, changes to these forces due to gestational age, environment or pathological condition will likely have an effect on the biomechanical stimuli experienced by the hip joint.

In summary, this research represents the first quantification of fetal membrane and uterine wall deformation, and provides novel predictions of contact forces and muscle forces generated during fetal movements. These results will be applied in a second set of FE models of fetal joints to investigate the local biomechanical stimuli induced by the muscles identified here. By repeating this approach over a large number of scans, it will be possible to determine the effect of gestational age and the restrictiveness of the uterus environment on the mechanical stimuli experienced/induced in the fetal skeleton. Therefore, this computational pipeline will enable us to identify environments which increase the risk of joint malformations, helping clinicians to consider interventions prenatally, to perform more intensive screening on at-risk infants after birth, or to prescribe suitable postnatal physiotherapy. This research may therefore inform future preventative measures for neonatal joint conditions such as DDH, thereby potentially reducing the risk of osteoarthritis in later life.
